# The Oncogenic and Immunological Roles of Apoptosis Antagonistic Transcription Factors in Human Tumors: A Pan-Cancer Analysis

**DOI:** 10.1155/2022/3355365

**Published:** 2022-10-12

**Authors:** Feng Lu, Juan Wu, Hongpeng Zou, Xin Yang, Yongbing Wu, Jianjun Xu

**Affiliations:** ^1^Department of Cardiothoracic Surgery, The Second Affiliated Hospital of Nanchang University, Nanchang, Jiangxi 330006, China; ^2^Department of Respiratory Diseases, The Second Affiliated Hospital of Nanchang University, Nanchang, Jiangxi 330006, China

## Abstract

**Background:**

Apoptosis-antagonizing transcription factor (AATF) participates in tumor progression in multiple cancer types. However, its role across cancers is not well understood.

**Methods:**

Data from The Cancer Genome Atlas (TCGA), Genotype-Tissue Expression (GTEx), Clinical Proteomic Tumor Analysis Consortium (CPTAC), and Human Protein Atlas (HPA) were used to analyze the multiomic roles of AATF in 33 tumor types, including gene and protein expression, survival prognosis, gene mutation, DNA methylation, protein phosphorylation, AATF coexpressed genes and their enrichment analysis, and immunological analysis.

**Results:**

In TCGA and GTEx databases, 31 tumors and their corresponding normal tissues had AATF expression data, and it was differentially expressed in 29 of them. AATF was elevated in 27 tumors, decreased in 2 tumors, and was a risk factor for overall survival (OS) in 8 tumors and a risk factor for disease-free survival (DFS) in 4 tumors. AATF expression levels in various cancer types were significantly correlated with the infiltration levels of cancer-associated fibroblasts, endothelial cells, CD4+ T cells, B cells, myeloid dendritic cells, eosinophils, and macrophages. The immune checkpoints PD-1, PD-L1, and CTLA4 were positively correlated with AATF expression in bladder urothelial carcinoma (BLCA), kidney chromophobe (KICH), and prostate adenocarcinoma (PRAD).

**Conclusion:**

In cancer, AATF expression is generally higher than that in normal tissue, and it is also associated with immunomodulation-related genes. AATF may be a risk factor for poor prognosis across cancers.

## 1. Introduction

Cancer is one of the main causes of death worldwide [1]. Tumors are mainly caused by molecular aberrations, including somatic mutations, copy number alterations, chromosomal rearrangements, transcriptional expression changes, and epigenetic variations [2]. The Cancer Genome Atlas (TCGA) is an applied platform for genome sequencing analysis of large samples of 33 cancers, which enables us to systematically analyze molecular aberrations in cancers by applying genomic technologies [3]. Pan-cancer studies are necessary for understanding cancer-generating systems.

As a natural barrier to cancer development, apoptosis regulates cell death and inhibits the growth of cancer cells [4]. Apoptosis removes potentially harmful cells, which can stop tumor growth [5]. Efficient elimination of cancer cells through programmed cell death or apoptosis is a mainstay and goal of clinical cancer therapy [6]. Apoptosis-antagonizing transcription factor (AATF), which binds to RNA polymerase II, is involved in transcriptional regulation, DNA damage response, cell cycle regulation, and apoptosis [7]. Jing et al. [8] found that compared to normal tissues, AATF expression is increased in Wilms' tumor, and it can also promote the proliferation, invasion, and migration of Wilms' tumors. Welcker et al. [9] showed that AATF inhibits p53-driven apoptosis in some tumor tissues in vivo and contributes to their proliferation and progression. Desantis et al. [10] reported that AATF can maintain tumor cell survival by controlling the autophagy response and endoplasmic reticulum stress. Based on the above studies, AATF plays a number of important roles in cancer development; for example, cancer cells are protected from apoptosis induction or autophagy, thus promoting cancer cell survival and cancer progression.

In the absence of a comprehensive description of AATF's molecular properties in human cancer, the role of AATF in pan-cancer research is currently unknown. In this study, we used multiple TCGA-based tools to comprehensively analyze the expression signature of AATF in different types of cancer for the first time. Additionally, various potential biological functions and common features of AATF across cancers were analyzed and verified to explore the potential molecular mechanism of AATF across cancers.

## 2. Method

### 2.1. Gene Expression Analysis

The AATF was entered into the “Gene_DE” module of the TIMER2.0 online tool (http://timer.comp-genomics.org/) to acquire AATF gene expression data in different tumors or specific tumor subtypes and adjacent normal tissues in TCGA database [11]. RNA-seq data in transcripts per million read (TPM) format for TCGA and Genotype-Tissue Expression (GTEx) were downloaded from the University of California, Santa Cruz (UCSC XENA; https://xena.ucsc.edu/) [12, 13]. The RNA-seq data in TPM format were analyzed and compared after log_2_ conversion and visualized using the “ggplot” package of R software. Unpaired samples *t*-test was used to compare the expression level of AATF between the normal and tumor groups; statistical significance was set at *P* < 0.05. Abbreviations for all tumor names can be found in the abbreviation table (Supplementary Table 1).

### 2.2. Protein Expression Analysis and Immunohistochemical (IHC) Staining Analyses

The University of Alabama at Birmingham Cancer Data Analysis Portal (UALCAN) (http://ualcan.path.uab.edu/analysis prot.html) is an interactive web resource for analyzing cancer omics data, enabling protein expression analysis on the Clinical Proteomic Tumor Analysis Consortium (CPTAC) dataset [14, 15]. The gene name AATF was entered into the “CPTAC” module of the UALCAN website to analyze and compare the expression levels of the total protein of AATF between cancer tissues and normal tissues. To verify the expression of AATF protein at the histological level, immunohistochemistry- (IHC-) based AATF protein expression maps in various tumors and corresponding normal tissues were downloaded from the Human Protein Atlas (HPA) database (http://www.proteinatlas.org/) [16].

### 2.3. Analysis of the Relationship between AATF Expression and Different Pathological Stages of Tumors

The relationship between RNA expression of AATF and tumor pathological stage was analyzed using the “Pathological Stage Plot” module of the GEPIA2 (http://gepia2.cancer-pku.cn/#analysis) tool [17]. Differences in RNA expression of AATF at different pathological stages of various tumors were visualized using violin plots. The relationship between AATF protein expression in the CPTAC database and eight tumor pathological stages was analyzed in UALCAN.

### 2.4. Survival Prognosis Analysis

Overall survival (OS) and disease-free survival (DFS) of AATF in all TCGA tumor patients were analyzed using the “Survival Analysis” module of GEPIA2, and survival maps were plotted.

### 2.5. Genetic Alteration Analysis

Genetic alteration analysis of AATF was performed by using “TCGA Pan-Cancer Atlas Studies” dataset in the cBio Cancer Genomics Portal (http://cbioportal.org) tool [18]. Use the “Summary of Cancer Types” submenu to visualize genetic alteration frequencies.

### 2.6. Promoter Methylation and Protein Phosphoprotein Analysis

Promoter methylation and protein phosphorylation levels of AATF between different cancers and corresponding normal tissues were analyzed using the UALCAN website. Data on promoter methylation were based on TCGA database. Data on protein phosphorylation were based on the CPTAC database.

### 2.7. Coexpression Heat Map, Enrichment Analysis, and PPI Network

Protein–protein interaction (PPI) networks were generated by the STRING website (https://string-db.org) [19]. The parameter settings are as follows: meaning of network edges select “evidence,” active interaction sources select “Experiments,” minimum required interaction score select “medium confidence 0.4,” and max number of interactors to show select “no more than 20 interactors.” Finally, the experimentally determined binding protein of AATF was obtained. The open-source bioinformatics software platform Cytoscape (version 3.8.2) was used to visualize molecular interaction networks [20]. The top 100 AATF-correlated genes in the datasets of all TCGA tumors were identified by using GEPIA2. The correlation of the top 10 AATF coexpressed genes with tumors was identified in TIMER2.0, and the results are shown as a heat map. Gene Ontology (GO) and Kyoto Encyclopedia of Genes and Genomes (KEGG) enrichment analyses were performed on AATF and the top 100 relevant genes downloaded from GEPIA2. Analysis was performed using the clusterProfiler R package (version 3.14.3). The ggplot2 R package (version 3.3.3) was used for visualization.

### 2.8. Immune Infiltration Analysis and Correlation Analysis of Immune Regulation-Related Genes

The association between AATF expression and immune infiltration in all TCGA tumors was analyzed using the “Immune-Gene” module of TIMER2.0. Cancer-associated fibroblasts, endothelial cells, B cells, CD4+ T cells, myeloid dendritic cells, macrophages, and eosinophils were selected for detailed analysis. To assess the relationship between AATF expression and immune checkpoint genes, the data on the correlation between AATF and immune checkpoint genes across cancers were analyzed from the “Immune Association” module of TIMER2.0 and downloaded. The ggplot2 R package (version 3.3.3) was used for visualization.

## 3. Result

### 3.1. Cancer and Normal Tissue Expression Levels of AATF

**Figure 1:**
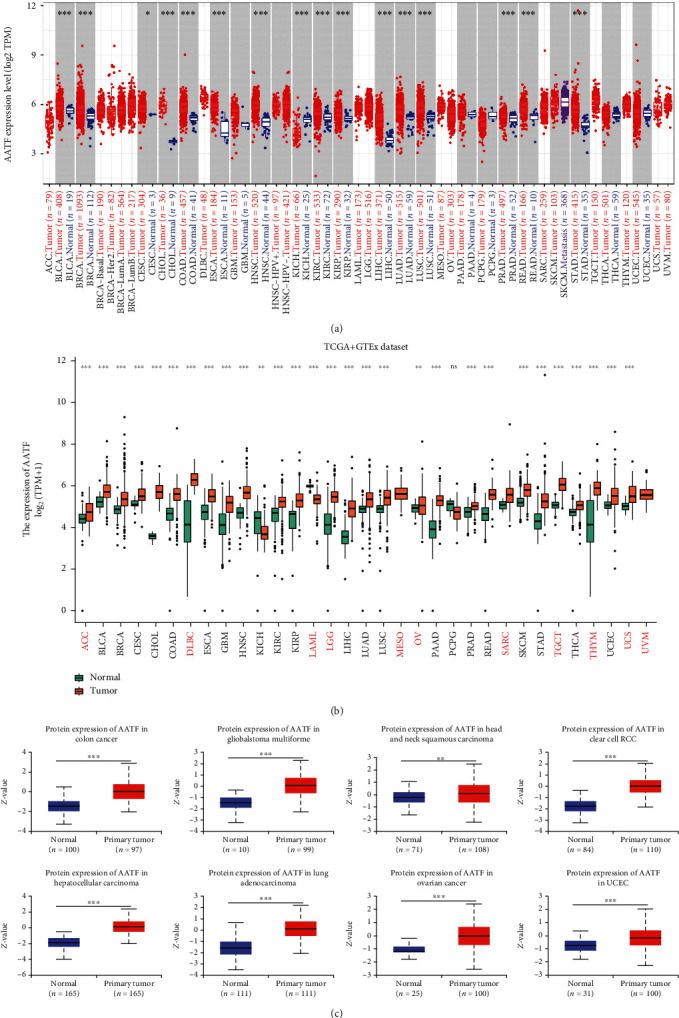
RNA and protein expression levels of AATF in human tumors and adjacent normal tissues (^∗^, *P* < 0.05; ^∗∗^, *P* < 0.01; ^∗∗∗^, *P* < 0.001; ns, no significance). (a) We used TIMER2.0 to analyze the RNA expression levels of AATF in different tumors. The gray background shows the expression of AATF in the tumor and adjacent normal tissues and compares them, and the white background shows the AATF expression in only tumor tissues. (b) AATF expression in different tumors and normal tissues after TCGA and GTEx using combined data. The red font represents tumors without normal tissue data in TCGA. (c) Protein data were extracted and analyzed using the CPTAC dataset, comparing the protein expression of AATF between cancer and normal tissues.

To clarify the expression level of AATF across cancers, we first analyzed the TIMER2.0 tool, and 21 of the 33 tumors had tumor and adjacent normal tissue data. Comparing AATF expression in tumors and adjacent normal tissues, AATF was elevated in 15 tumors and decreased in 1 tumor (*P* < 0.05). Because there were no adjacent normal tissue controls, 10 tumors only showed AATF expression levels in tumors (Figure 1(a)). Next, TCGA and GTEx data were combined to compare the expression of AATF in pan-cancer and normal tissues. After combining the data, 2 of the 33 tumors still had no normal tissues to compare. By comparing the expression of AATF in 31 tumors and normal tissues, it was found that AATF was different in 29 tumors. It was elevated in 27 tumors and decreased in 2 tumors (*P* < 0.05, Figure 1(b)).

### 3.2. Protein Expression Analysis Data and Immunohistochemical Staining of AATF

**Figure 2:**
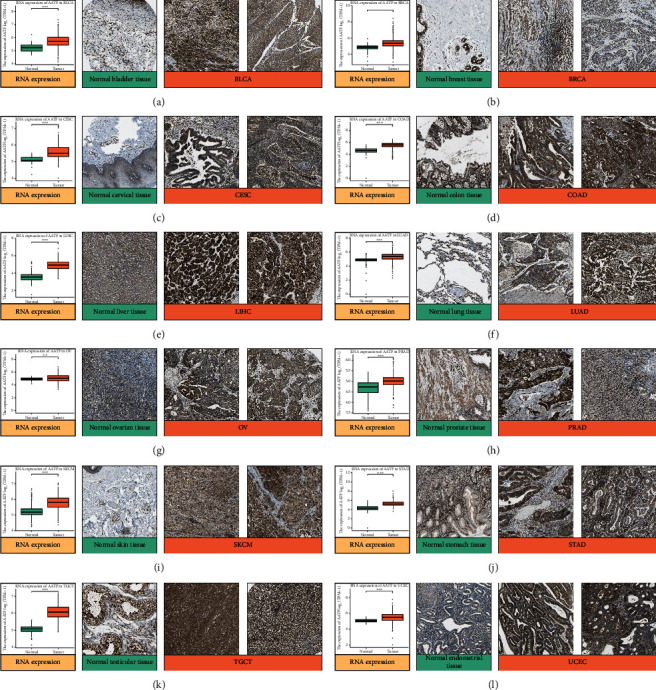
RNA expression of AATF in 12 tumor and normal tissues and compare the immunohistochemical images of AATF in these 12 tumor tissues and normal tissues in the HPA database (^∗^, *P* < 0.05; ^∗∗^, *P* < 0.01; ^∗∗∗^, *P* < 0.001): (a) normal bladder tissue vs. BLCA; (b) normal breast tissue vs. BRCA; (c) normal cervical tissue vs. CESC; (d) normal colon tissue vs. COAD; (e) normal liver tissue vs. LIHC; (f) normal lung tissue vs. LUAD; (g) normal ovarian tissue vs. OV; (h) normal prostate tissue vs. PRAD; (i) normal skin tissue vs. SKCM; (j) normal stomach tissue vs. STAD; (k) normal testicular tissue vs. TGCT; (l) normal endometrial tissue vs. UCEC.

Extraction and analysis of protein data were performed using the CPTAC dataset, and 10 tumors in the database had AATF protein expression-related data. Comparing the protein expression of AATF between cancer and normal tissues, AATF was elevated in 8 of the 10 tumors (*P* < 0.05, Figure 1(c)). The immunohistochemical results downloaded from the HPA database were analyzed and compared with the AATF gene expression data from TCGA+GTEx database. We found consistent analysis across 12 tumors, with AATF being upregulated in all 12 tumors (Figure 2).

### 3.3. AATF Expression Levels in Different Stages

**Figure 3:**
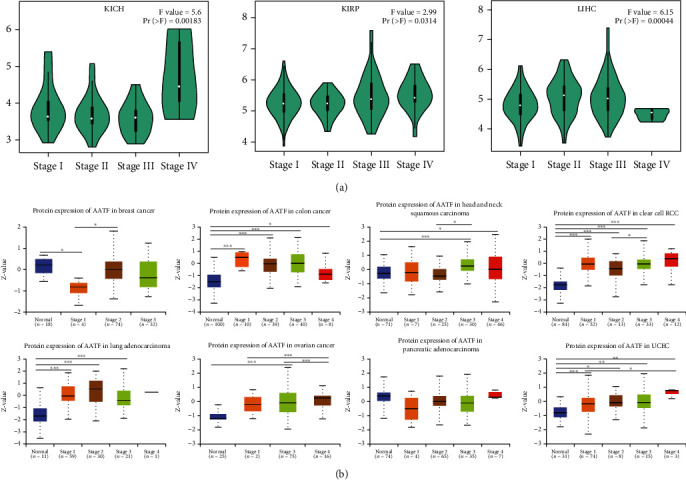
(a) TCGA database was used to analyze the relationship between the RNA expression of AATF and the pathological stage of KICH, KIRP, and LIHC. (b) The relationship between AATF protein expression and 8 tumor pathological stages was analyzed in the CPTAC database.

TCGA database was used to analyze the relationship between AATF RNA expression and 33 tumor pathological stages, among which kidney chromophobe (KICH), kidney renal papillary cell carcinoma (KIRP), and liver hepatocellular carcinoma (LIHC) were significantly different in each pathological stage (*P* < 0.05, Figure 3(a)). The relationship between AATF protein expression in the CPTAC database and the pathological stage of 8 tumors was analyzed on the UALCAN website, of which 7 tumors had differences in different pathological stages, including breast invasive carcinoma (BRCA), colon adenocarcinoma (COAD), kidney renal clear cell carcinoma (KIRC), head and neck squamous cell carcinoma (HNSC), lung adenocarcinoma (LUAD), ovarian serous cystadenocarcinoma (OV), and uterine corpus endometrial carcinoma (UCEC) (*P* < 0.05, Figure 3(b)).

### 3.4. Survival Prognostic Analysis Data

**Figure 4:**
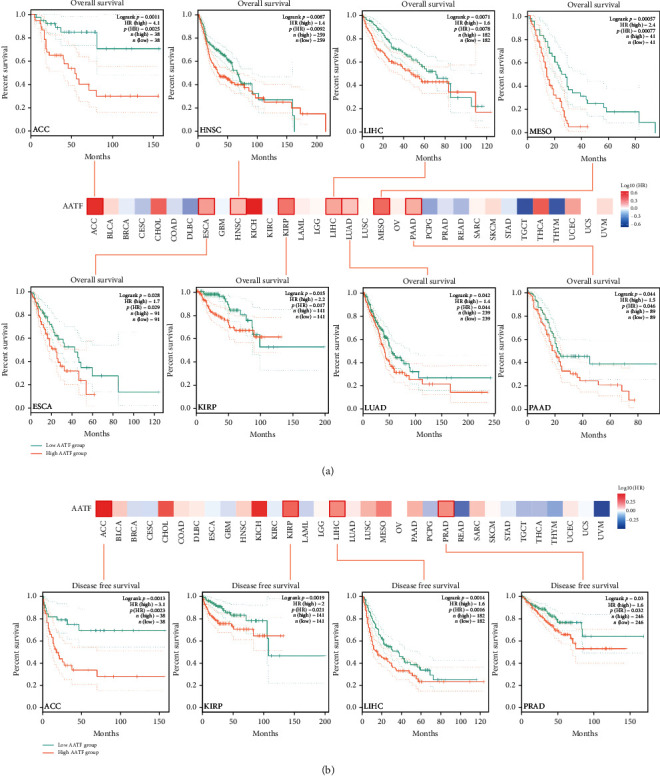
Survival prognostic analysis. (a) Overall survival and (b) disease-free survival analysis of different tumors in TCGA by AATF gene expression. Survival maps and Kaplan–Meier curves for statistically significant results are shown.

The OS and DFS of AATF in all TCGA tumor patients were analyzed using GEPIA2. Elevated AATF expression was found to be a risk factor for OS in 8 tumors (adrenocortical carcinoma (ACC) (HR = 4.1, *P* = 0.025), esophageal carcinoma (ESCA) (HR = 1.7, *P* = 0.029), HNSC (HR = 1.4, *P* = 0.0092), KIRP (HR = 2.2, *P* = 0.017), LIHC (HR = 1.6, *P* = 0.0078), LUAD (HR = 1.4, *P* = 0.044), mesothelioma (MESO) (HR = 2.4, *P* = 0.00077), and pancreatic adenocarcinoma (PAAD) (HR = 1.5, *P* = 0.046)) (Figure 4(a)). Elevated AATF expression was also a risk factor for DFS in 4 tumors, including ACC, KIRP, LIHC, and prostate adenocarcinoma (PRAD) (ACC (HR = 3.1, *P* = 0.0023), KIRP (HR = 2, *P* = 0.021), LIHC (HR = 1.6, *P* = 0.0016), and PRAD (HR = 1.6, *P* = 0.032)) (Figure 4(b)).

### 3.5. Genetic Alteration Analysis Data

**Figure 5:**
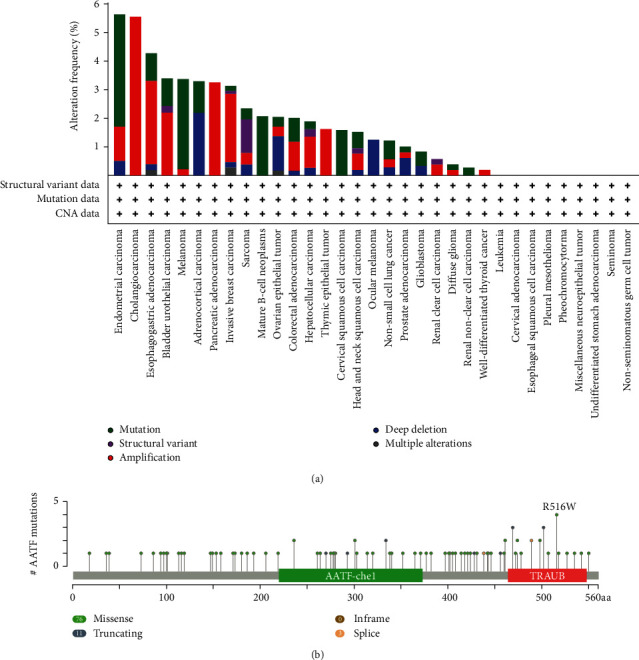
The mutational feature of AATF in different tumors of TCGA was analyzed using the cBioPortal tool. (a) Mutation type and (b) mutation site are shown.

The mutational features of AATF in different tumors in TCGA were analyzed using the cBioPortal tool. The analysis found that the frequency of AATF mutations was the highest in UCEC, with 5.63% (33/583) of UCEC cases harboring genetic variations (Figure 5(a)). We also discovered and visualized mutation sites of AATF, as shown in Figure 5(b). Missense mutations were the most common type of mutation and were randomly distributed within AATF; among them, the R516 W site was the most frequently mutated site, with 4 missense mutations occurring at this site.

### 3.6. Promoter Methylation and Protein Phosphorylation Data

**Figure 6:**
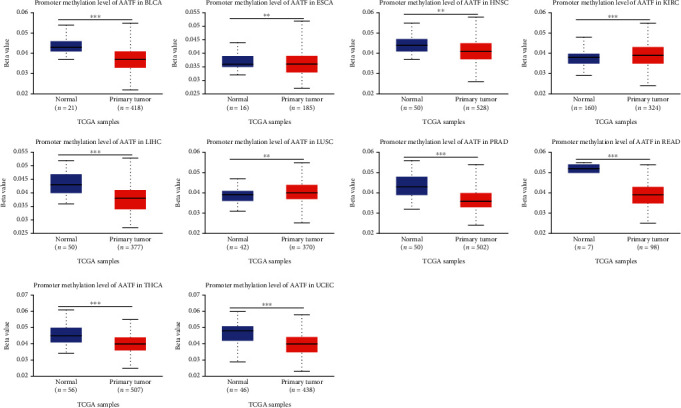
The promoter methylation level of AATF in the different tumors.

**Figure 7:**
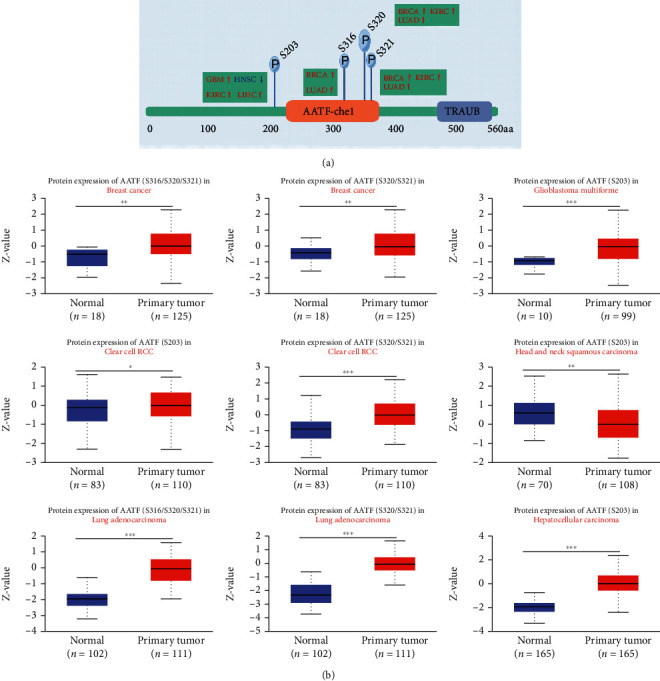
Phosphorylation analysis of the AATF protein in different tumors. (a) Schematic diagram of the phosphorylation protein site of AATF. (b) Phosphorylation levels and phosphorylation sites of AATF protein in tumor and normal tissues (^∗^, *P* < 0.05; ^∗∗^, *P* < 0.01; ^∗∗∗^, *P* < 0.001).

Promoter methylation is closely related to the occurrence and progression of cancer [21]. After analyzing the promoter methylation levels of AATF between different cancers and corresponding normal tissues using data from TCGA on the UALCAN website, it was found that, compared with normal tissues, AATF promoter methylation levels were significantly elevated in KIRC and lung squamous cell carcinoma (LUSC) (*P* < 0.05), whereas AATF promoter methylation levels were significantly decreased in 8 tumors, including bladder urothelial carcinoma (BLCA), ESCA, HNSC, LIHC, PRAD, rectum adenocarcinoma (READ), thyroid carcinoma (THCA), and UCEC (*P* < 0.05, Figure 6). Protein phosphorylation is also inextricably linked to cancer [22]. We also compared differences in AATF protein phosphorylation levels between normal and primary tumor tissues. The protein phosphorylation level of AATF at 4 sites, including S203, S316, S320, and S321, was changed in six tumors (BRCA, glioblastoma multiforme (GBM), LIHC, HNSC, KIRC, and LUAD) with data in the CPTAC database on the UALCAN website (Figure 7(a)). Compared with normal tissues, except for HNSC, which was decreased at the S203 site, the other five tumors had increased phosphorylation levels of AATF at the corresponding phosphorylation sites (Figure 7(b)).

### 3.7. AATF Coexpressed Genes and Enrichment Analysis Results and PPI Network

**Figure 8:**
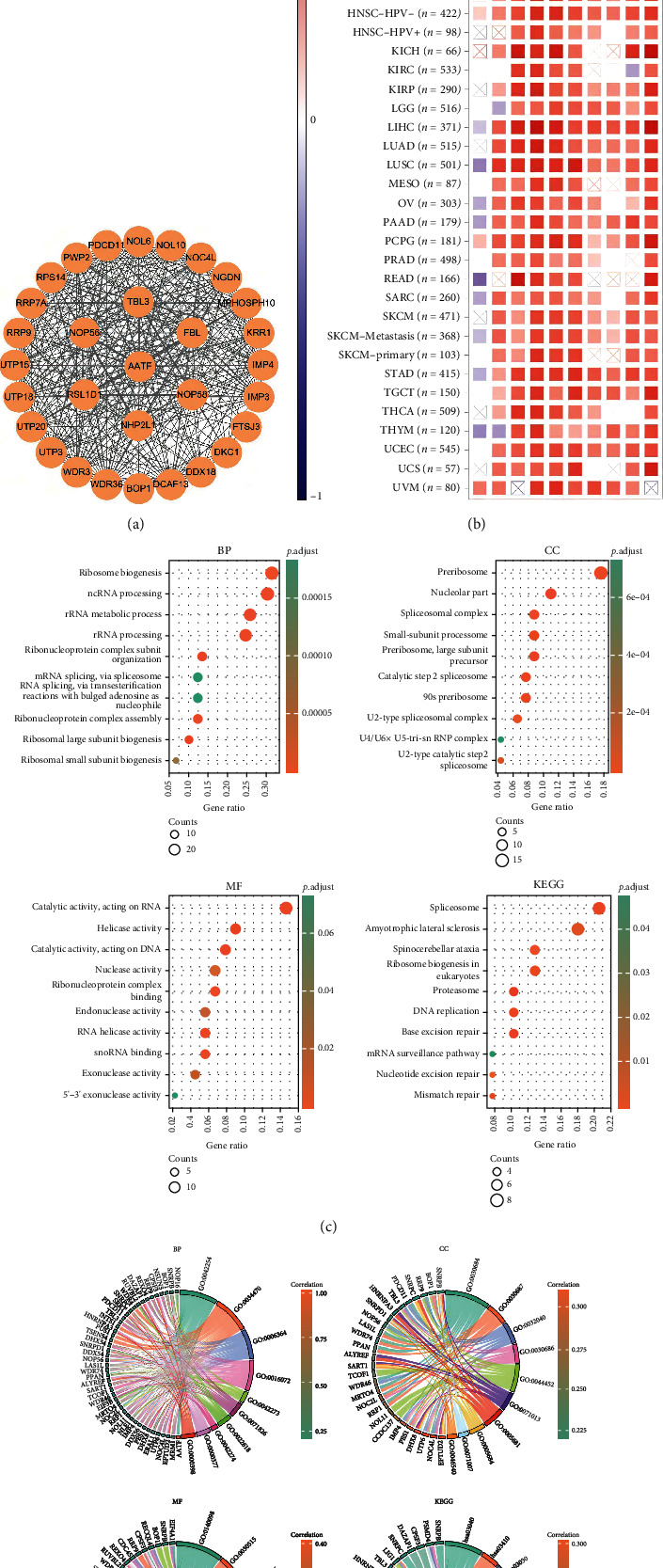
AATF-related gene enrichment analysis. (a) The protein–protein interaction network was analyzed via the STRING online resource. (b) A heat map of expression correlations of the top 10 AATF coexpressed genes in tumors was drawn by TIMER2. The top 100 interacting genes of GEPIA2 subjected to KEGG and GO pathway analysis are shown as (c) bubble plots and (d) chord diagrams. (For detailed results in (c) and (d), refer to Supplementary Table 2).

Using the STRING website, 20 experimentally detected AATF-related genes were analyzed, and the PPI network was created and visualized using Cytoscape (Figure 8(a)). A heat map of expression correlations between AATF and representative genes of the top AATF-related genes in TCGA project identified by GEPIA2 was plotted in TIMER2.0 (Figure 8(b)). AATF and the top 100 coexpressed genes of GEPIA2 were subjected to GO and KEGG pathway analysis (Figures 8(c) and 8(d)). The GO results showed that these genes were mainly enriched in the functional categories “ribosome biogenesis,” “catalytic activity, acting on RNA,” and “preribosome.” The KEGG results showed that these genes were mainly enriched in “spliceosome,” “base excision repair,” “DNA replication,” “proteasome,” and “mismatch repair.”

### 3.8. Correlation Analysis of AATF and Immune Cell Infiltration and the Expression of Immunomodulation-Related Genes

**Figure 9:**
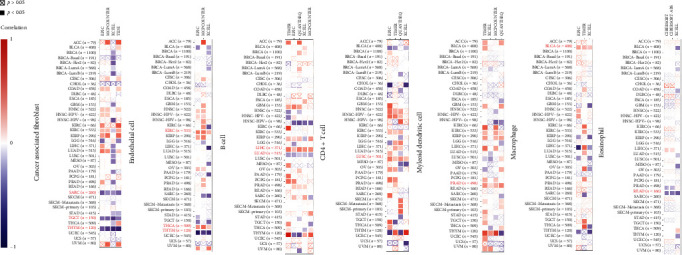
The correlation between the AATF expression level and immune infiltration of cancer-associated fibroblasts, endothelial cells, B cells, CD4+ T cells, myeloid dendritic cells, macrophages, and eosinophils.

Tumor-infiltrating immune cells are an important part of the tumor microenvironment and are closely related to the occurrence, progression, and metastasis of tumors [23, 24]. Therefore, we explored the correlation of AATF expression levels with immune infiltration of cancer-associated fibroblasts, endothelial cells, CD4+ T cells, B cells, myeloid dendritic cells, eosinophils, and macrophages (Figure 9). We found that the expression of AATF was negatively correlated with the infiltration of cancer-associated fibroblasts in sarcoma (SARC), testicular germ cell tumors (TCGT), and thymoma (THYM). The expression of AATF was positively correlated with endothelial cell infiltration in KIRC and THCA and negatively correlated in THYM. The expression of AATF was positively correlated with B cell infiltration in LIHC and negatively correlated in LUAD. CD4+ T cell infiltration in LUSC, myeloid dendritic cell infiltration in PRAD, and macrophage infiltration in BLCA were all positively correlated with the expression of AATF. In READ, eosinophil infiltration was negatively correlated with AATF expression.

**Figure 10:**
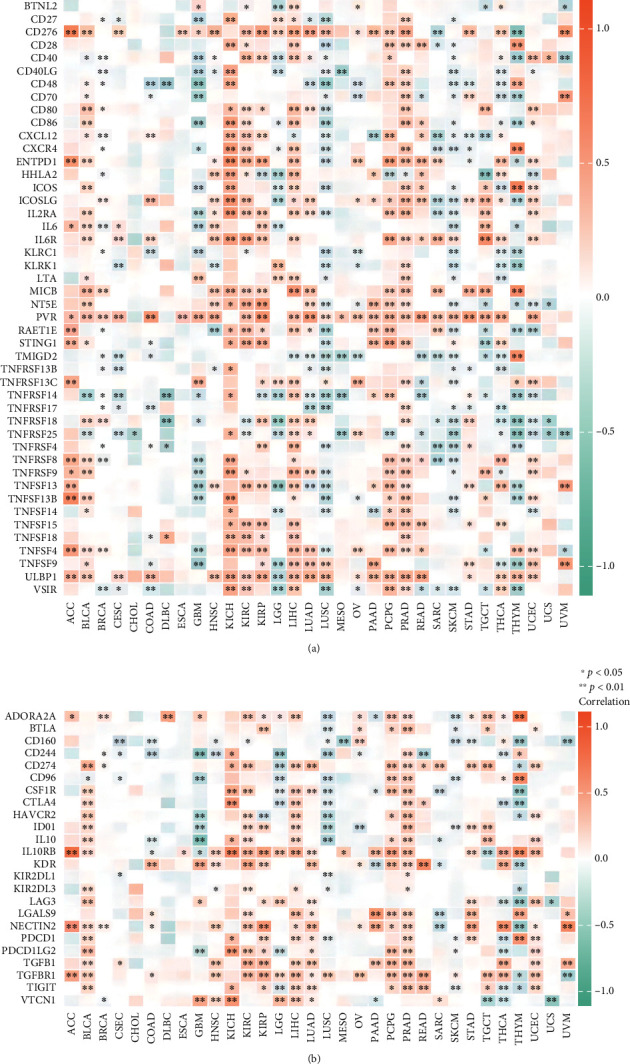
The correlation between AATF and immunoregulation-related genes. (a) A heat map represents the correlation between AATF expression and immunostimulatory genes. (b) A heat map represents the correlation between AATF expression and immunosuppressive status-related genes.

Tumors can evade immune responses by exploiting immune checkpoint genes such as PD-1 and CTLA-4 [25]. To closely estimate the association between AATF expression and the tumor microenvironment in the pan-cancer dataset, we further investigated the relationship between AATF expression and immunomodulation-related genes, including immune checkpoint, immunostimulatory, and immunosuppressive genes. Notably, we observed that AATF expression was positively correlated with most of the immune regulation-related genes in KICH, LIHC, and PRAD (Figure 10). To better show the significant results, we combine the significant results in Figures 9 and 10 together and plotted in Supplementary Figure 1.

## 4. Discussion

AATF was first reported in 1999 [26]. AATF is involved in the regulation of cell proliferation, DNA damage response, apoptosis, and cell cycle arrest [7]. There is still a need for further study to determine whether AATF can contribute to the pathogenesis of different types of tumors through common molecular mechanisms. We were unable to locate any studies analyzing the whole pan-cancer from the perspective of AATF through our literature search. Therefore, starting from gene expression, survival prognosis, promoter methylation, protein phosphorylation, gene mutation, immune infiltration, and immunomodulation-related gene expression, we conducted a comprehensive detection and analysis of the AATF gene in a total of 33 tumors in TCGA and CPTAC databases. In TCGA and GTEx databases, 31 tumors and their corresponding normal tissues had AATF expression data, and it was different in 29 of them compared with normal tissues. It was elevated in 27 tumors and decreased in 2 tumors. This suggests that AATF is closely linked to most cancers.

Apoptosis is prevented by AATF, which regulates gene transcription and cell proliferation [27]. AATF has been shown to be overexpressed in a variety of cancer tissues, such as breast cancer, multiple myeloma, leukemia, lung cancer, hepatocellular carcinoma, and head and neck squamous cell carcinoma, and its level increases during disease progression [9, 10, 28–33]. AATF is also a regulator of tumor cell survival and tumor progression, and studies have found that silencing AATF promotes apoptosis in breast cancer cells [32, 34]. Tan et al. [35] found that AATF is overexpressed in human bladder cancer and promotes cancer progression through its regulation of cyclin E and survivin, suggesting that a potential marker and therapeutic target for bladder cancer can be identified by using AATF. Kumar et al. [30] found that MCP1 can be upregulated by AATF through STAT3 and become a key factor in hepatocellular carcinoma development. Additionally, reducing AATF levels significantly reduced hepatocellular carcinoma cell oncogenic properties, such as proliferation, migration, and angiogenesis, and reduced cell death. According to the current study, AATF expression in most tumors was higher than that in normal tissues. Clearly, AATF plays a critical role in the progression of cancer, possibly by preventing apoptosis induction and promoting cell survival.

In cancer research, survival analysis is a critical analytical index for assessing disease prognosis. Liu et al. [36] found that elevated AATF leads to poor prognosis in hepatocellular carcinoma patients. Most neuroblastoma patients express AATF gene amplification, and a high level of AATF is linked to a poor prognosis and reduced survival [37]. In our research, elevated AATF expression was a risk factor for OS in 8 tumors and a risk factor for DFS in 4 tumors. AATF is also closely related to tumor survival and prognosis. However, how AATF affects tumor survival and prognosis requires further research. As a result of AATF depletion, tumor cells are sensitive to anticancer drugs, suggesting that AATF might be a potential therapeutic target [38].

Gene mutation is one of the main causes of tumorigenesis. Sharma et al. [39] reported a novel oncogenic mutant AATF protein that was discovered in an aberrant AATF transcriptome. They used stem cells as a prototype model in a follow-up study to explore whether overexpression of APOBEC3G affects AATF gene expression in these cells and genes involved in oncogenic transformation [40]. They found that APOBEC3G binds AATF mRNA within its third exon and promotes the translation of a truncated 23 kDa product; in turn, it has the intrinsic ability to mediate oncogenic transformation induced by APOBEC3G in such cells [40]. However, the mechanism of AATF mutation is unclear. Only the mutational signature of AATF and the mutational sites of AATF in different tumors of TCGA are shown in this study.

In many tumors, promoter methylation is essential for regulating tumor gene expression. According to the study, AATF promoter methylation levels were significantly lower in 8 tumor tissues than in normal tissues, potentially correlating with the high expression of AATF in these tumors. An important posttranslational process that modulates protein activity and interactions is phosphorylation. It has been demonstrated that phosphorylation of AATF enhances its binding to p53, thereby regulating p53 activity [41]. We identified 4 phosphorylation sites, S203, S316, S320, and S321, in 6 tumors with significantly changed AATF protein phosphorylation levels in the CPTAC database. A greater understanding of the possible role of AATF promoter methylation and protein phosphorylation in tumorigenesis is needed.

This study revealed the relationship between AATF and tumor-infiltrating immune cells and investigated the immune status of cancer patients by detecting the expression of AATF. AATF expression levels in various cancer types were significantly correlated with the infiltration levels of cancer-associated fibroblasts, endothelial cells, CD4+ T cells, B cells, myeloid dendritic cells, eosinophils, and macrophages. In cancer patients, choosing the right individualized immunotherapy strategy is especially important [42, 43]. In recent years, immune checkpoint blockade therapy has changed the landscape of cancer treatment and has become one of the most important immunotherapies in the treatment of cancer [44]. We explored the immune checkpoints associated with AATF and found that AATF expression was associated with immune checkpoint genes in many tumors. The results suggest that the function of immune cells regulated by AATF is closely related to the occurrence and development of tumors. Major immune checkpoints include PD-1, PD-L1, and CTLA4 [45]. In the present study, we found that the expression levels of PD-1, PD-L1, and CTLA4 in BLCA, KICH, and PRAD were significantly positively correlated with AATF. Across a variety of cancers, the expression of AATF was highly correlated with immune infiltration and immune checkpoint markers, which indicates that AATF may be a target for immunotherapy.

However, this study also has some limitations. First, this research was based on data obtained from a public database, without further validation of our findings at the cellular and animal levels. Furthermore, the pathophysiological mechanisms underlying the results have not been systematically and deeply explored. Therefore, further in vitro and in vivo studies are required to elucidate the oncogenic mechanism of AATF and its potential as a therapeutic target.

## 5. Conclusion

In conclusion, an in-depth analysis of the pan-cancer impact of AATF was performed, including gene and protein expression, survival prognosis, gene mutation, promoter methylation, protein phosphorylation, AATF coexpressed genes and their enrichment analysis, immune cell infiltration, and the expression of immunomodulation-related genes. We found that the expression of AATF was higher in cancer than in normal tissues, and it was also associated with genes related to immune regulation. This study helps us to understand the role of AATF in tumorigenesis from the perspective of clinical tumor samples. AATF may be a risk factor for poor prognosis across cancers.

## Data Availability

All data can be downloaded from the online public data TCGA, GTEx, CPTAC, and HPA.

## Supplementary Materials

Supplementary Materials Supplementary Figure 1: combination of the significant results in Figures 9 and 10 together. CAF: cancer-associated fibroblasts; Endo: endothelial cells; mDC: myeloid dendritic cells; Eos: eosinophils; Mac: macrophages. Supplementary Table 1: abbreviations. Supplementary Table 2: GO and KEGG enrichment analysis of AATF and the top 100 coexpressed genes in Figures 8(c) and 8(d).
