# Supplementary material for: The Oncogenic and Immunological Roles of Apoptosis Antagonistic Transcription Factors in Human Tumors: A Pan-Cancer Analysis
Source: Oxid Med Cell Longev. 2022 Oct 12;2022:3355365. doi: 10.1155/2022/3355365 (PMC9581705; doi:10.1155/2022/3355365)
Supplement: Supplementary Materials — Supplementary Figure 1: combination of the significant results in Figures 9 and 10 together. CAF: cancer-associated fibroblasts; Endo: endothelial cells; mDC: myeloid dendritic cells; Eos: eosinophils; Mac: macrophages. Supplementary Table 1: abbreviations. Supplementary Table 2: GO and KEGG enrichment analysis of AATF and the top 100 coexpressed genes in Figures 8(c) and 8(d). [file 3355365.f1.docx]

**Supplementary Figure 1**


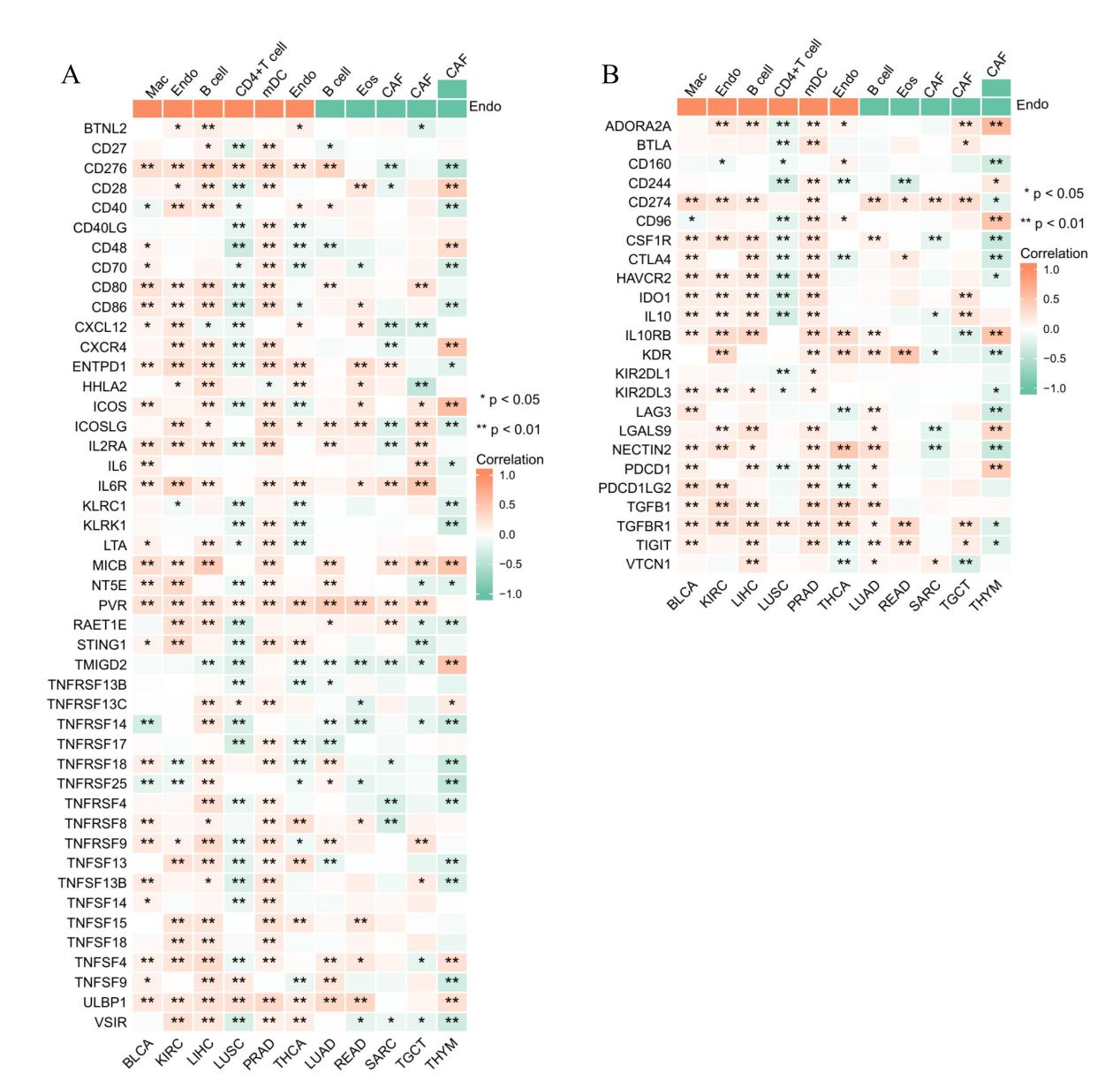


**Supplementary Figure 1: Combination of the significant results in Figure 9 and Figure 10 together.** CAF:cancer-associated fibroblasts, Endo: endothelial cells, mDC: myeloid dendritic cells, Eos: eosinophils, Mac: macrophages.

**Supplementary Table 1.** Abbreviations Table

| Abbreviations | Full name |
| --- | --- |
| AATF | Apoptosis antagonizing transcription factor |
| ACC | Adrenocortical carcinoma |
| BLCA | Bladder Urothelial Carcinoma |
| BRCA | breast invasive carcinoma |
| COAD | colon adenocarcinoma |
| CPTAC | Clinical proteomic tumor analysis consortium |
| DFS | disease-free survival |
| ESCA | esophageal carcinoma |
| GBM | glioblastoma multiforme |
| GO | Gene Ontology |
| GTEx | the Genotype-Tissue Expression |
| HNSC | head and neck squamous cell carcinoma |
| HPA | Human Protein Atlas |
| IHC | immunohistochemical |
| KEGG | Kyoto Encyclopedia of Genes and Genomes |
| KICH | kidney chromophobe |
| KIRC | kidney renal clear cell carcinoma |
| KIRP | kidney renal papillary cell carcinoma |
| LIHC | liver hepatocellular carcinoma |
| LUAD | Lung adenocarcinoma |
| LUSC | Lung squamous cell carcinoma |
| MESO | mesothelioma |
| OS | overall survival |
| OV | ovarian serous cystadenocarcinoma |
| PAAD | pancreatic adenocarcinoma |
| PPI | Protein‒protein interaction |
| PRAD | Prostate adenocarcinoma |
| READ | Rectum adenocarcinoma |
| SARC | Sarcoma |
| TCGA | The Cancer Genome Atlas |
| TCGT | Testicular Germ Cell Tumors |
| THCA | Thyroid carcinoma |
| THYM | Thymoma |
| TPM | transcripts per million reads |
| UALCAN | The University of Alabama at Birmingham Cancer data analysis Portal |
| UCEC | uterine corpus endometrial carcinoma |
| UCSC Xena | the University of California Santa Cruz Xena |

**Supplementary Table 2.** GO and KEGG enrichment analysis of AATF and the top 100 coexpressed genes in Figure 8C and Figure 8D

| Ontology | ID | Description | GeneRatio | BgRatio | P value | p.adjust | q value |
| --- | --- | --- | --- | --- | --- | --- | --- |
| BP | GO:0042254 | ribosome biogenesis | 28/89 | 297/18670 | 5.49e-29 | 5.50e-26 | 4.20e-26 |
| BP | GO:0034470 | ncRNA processing | 27/89 | 384/18670 | 1.73e-24 | 8.67e-22 | 6.63e-22 |
| BP | GO:0006364 | rRNA processing | 22/89 | 214/18670 | 1.38e-23 | 4.62e-21 | 3.53e-21 |
| BP | GO:0016072 | rRNA metabolic process | 23/89 | 253/18670 | 2.10e-23 | 5.25e-21 | 4.02e-21 |
| BP | GO:0042273 | ribosomal large subunit biogenesis | 9/89 | 71/18670 | 4.91e-11 | 9.85e-09 | 7.53e-09 |
| BP | GO:0071826 | ribonucleoprotein complex subunit organization | 12/89 | 291/18670 | 1.34e-08 | 2.23e-06 | 1.71e-06 |
| BP | GO:0022618 | ribonucleoprotein complex assembly | 11/89 | 277/18670 | 8.25e-08 | 1.18e-05 | 9.03e-06 |
| BP | GO:0042274 | ribosomal small subunit biogenesis | 6/89 | 67/18670 | 7.82e-07 | 9.79e-05 | 7.49e-05 |
| BP | GO:0000377 | RNA splicing, via transesterification reactions with bulged adenosine as nucleophile | 11/89 | 379/18670 | 1.85e-06 | 1.82e-04 | 1.39e-04 |
| BP | GO:0000398 | mRNA splicing, via spliceosome | 11/89 | 379/18670 | 1.85e-06 | 1.82e-04 | 1.39e-04 |
| CC | GO:0030684 | preribosome | 16/91 | 76/19717 | 9.17e-23 | 1.83e-20 | 1.23e-20 |
| CC | GO:0030687 | preribosome, large subunit precursor | 8/91 | 23/19717 | 6.96e-14 | 6.93e-12 | 4.65e-12 |
| CC | GO:0032040 | small-subunit processome | 8/91 | 38/19717 | 6.57e-12 | 4.35e-10 | 2.93e-10 |
| CC | GO:0030686 | 90S preribosome | 7/91 | 31/19717 | 8.47e-11 | 4.22e-09 | 2.83e-09 |
| CC | GO:0044452 | nucleolar part | 10/91 | 178/19717 | 9.54e-09 | 3.80e-07 | 2.55e-07 |
| CC | GO:0071013 | catalytic step 2 spliceosome | 7/91 | 87/19717 | 1.53e-07 | 5.07e-06 | 3.40e-06 |
| CC | GO:0005681 | spliceosomal complex | 8/91 | 185/19717 | 2.26e-06 | 6.42e-05 | 4.31e-05 |
| CC | GO:0005684 | U2-type spliceosomal complex | 6/91 | 90/19717 | 3.73e-06 | 9.28e-05 | 6.23e-05 |
| CC | GO:0071007 | U2-type catalytic step 2 spliceosome | 4/91 | 30/19717 | 1.06e-05 | 2.35e-04 | 1.58e-04 |
| CC | GO:0046540 | U4/U6 x U5 tri-snRNP complex | 4/91 | 42/19717 | 4.16e-05 | 7.52e-04 | 5.05e-04 |
| MF | GO:0140098 | catalytic activity, acting on RNA | 13/89 | 386/17697 | 6.50e-08 | 1.33e-05 | 1.18e-05 |
| MF | GO:0030515 | snoRNA binding | 5/89 | 26/17697 | 1.74e-07 | 1.78e-05 | 1.57e-05 |
| MF | GO:0004386 | helicase activity | 8/89 | 163/17697 | 1.64e-06 | 1.12e-04 | 9.87e-05 |
| MF | GO:0003724 | RNA helicase activity | 5/89 | 78/17697 | 4.54e-05 | 0.002 | 0.002 |
| MF | GO:0043021 | ribonucleoprotein complex binding | 6/89 | 133/17697 | 5.61e-05 | 0.002 | 0.002 |
| MF | GO:0140097 | catalytic activity, acting on DNA | 7/89 | 213/17697 | 9.91e-05 | 0.003 | 0.003 |
| MF | GO:0004519 | endonuclease activity | 5/89 | 127/17697 | 4.51e-04 | 0.013 | 0.012 |
| MF | GO:0004518 | nuclease activity | 6/89 | 205/17697 | 5.87e-04 | 0.015 | 0.013 |
| MF | GO:0004527 | exonuclease activity | 4/89 | 81/17697 | 7.40e-04 | 0.017 | 0.015 |
| MF | GO:0008409 | 5'-3' exonuclease activity | 2/89 | 17/17697 | 0.003 | 0.061 | 0.054 |
| KEGG | hsa03040 | Spliceosome | 8/39 | 151/8076 | 4.67e-07 | 2.01e-05 | 1.38e-05 |
| KEGG | hsa03410 | Base excision repair | 4/39 | 33/8076 | 1.72e-05 | 3.51e-04 | 2.40e-04 |
| KEGG | hsa03030 | DNA replication | 4/39 | 36/8076 | 2.45e-05 | 3.51e-04 | 2.40e-04 |
| KEGG | hsa03050 | Proteasome | 4/39 | 46/8076 | 6.55e-05 | 7.04e-04 | 4.83e-04 |
| KEGG | hsa03430 | Mismatch repair | 3/39 | 23/8076 | 1.72e-04 | 0.001 | 8.73e-04 |
| KEGG | hsa03008 | Ribosome biogenesis in eukaryotes | 5/39 | 111/8076 | 1.78e-04 | 0.001 | 8.73e-04 |
| KEGG | hsa05017 | Spinocerebellar ataxia | 5/39 | 143/8076 | 5.75e-04 | 0.004 | 0.002 |
| KEGG | hsa03420 | Nucleotide excision repair | 3/39 | 47/8076 | 0.001 | 0.008 | 0.005 |
| KEGG | hsa05014 | Amyotrophic lateral sclerosis | 7/39 | 364/8076 | 0.002 | 0.008 | 0.005 |
| KEGG | hsa03015 | mRNA surveillance pathway | 3/39 | 97/8076 | 0.011 | 0.047 | 0.032 |
